# Annotations

**Published:** 1911-09-09

**Authors:** 


### ANNOTATIONS.

### A New Capital for Australia.

At the offices of the High Commissioner for the Commonwealth of Australia may now be seen a plaster model of the site for the proposed Federal capital city. This is being shown in connection with an open competition for designs and plans for the building of the city. The competition is, we believe, the first to be organised for designs on such a large scale, and is open to the whole world. The prizes offered are £1,750, £750, and £500, and the competing designs must be sent to Melbourne by the end of January next. The proposed city will be the permanent seat of the Federal Government, where all Commonwealth legislation will be enacted. For the purposes of the design the population is to be taken at 25,000 and the plans are to embody all kinds of public buildings, factories, colleges, barracks, and hospitals with parks, ornamental waters, and last, but not least, stadium. Here, at least, is an opportunity on a magnificent scale to carry out the principles of town-planning which have of recent years been so prominently discussed. Here, on an unhampered site in New South Wales, about seventy miles from the coast, is an unrivalled field for the display of architectural beauty and economic foresight. Twenty-five years ago, when the streets of Kuala Lumpur were being laid out, the President of the Sanitary Board endeavoured to look a hundred years ahead, and the result has been to the great and lasting benefit of the capital of the Federated Malay States. The builders of the new city in Australia will be comparatively free and unfettered in their task, and there is no reason to fear that the officials of sanitary reform will find that they are crying in the wilderness on this occasion.

### Syncope Followed by Drowning.

Syncope followed by drowning was the verdict given in the case of a young man described as a "scoutmaster," who after returning from a fatiguing march went for a bathe in a river and almost immediately sank. Deaths by drowning in the case of good swimmers often present some degree of mystery. The popular view is that the swimmers are seized with "cramp," but where the cramp is thought to occur is not quite clear. The "cramp" with which most of us are familiar is in the muscles of the calf of the leg. Painful though the affection is for a short period, it is doubtful whether it would so disturb a good swimmer as to paralyse his powers of self-preservation. It is far more likely that the so-called "cramp" is something of the nature of angina pectoris. This is especially likely to be the case when the water is cold. The first attack of severe cardiac pain in those who eventually succumb to angina pectoris is not uncommonly associated with the action of cold upon the surface of the body. Pain which will incapacitate a man from walking or even standing would undoubtedly deprive him of the power to remain on the surface of water. In the case referred to above there had been a fatiguing march before the accident in the water occurred. Excessive exercise may undoubtedly predispose to attacks of cardiac pain. Attacks of pain following this cause are as a rule comparatively slight, but should there be some organic affection present with which cardiac pain is likely sooner or later to be associated, the combination of fatigue, application of cold, and some morbid condition would readily explain the fatal event. In these cases there is probably most often some organic affection of the heart, such as disease of the cardiac muscle, in which there is a liability to sudden death.

### "Aërocoly" in Ill-thriven Children.

Many marasmic children, while very thin as to their limbs and thorax, present a remarkable distension of the abdomen which might be mistaken for comparative fatness, so that the erroneous impression that plenty of food is being absorbed and made use of might arise, when really the child is being insufficiently nourished. It is often stated in a general way that this abdominal distension of marasmic children is due in many instances to tuberculous mesenteric glands (tabes mesenterica), but that this is by no means always or commonly the case has been shown by many autopsies, and another explanation of the abdominal distension has been brought forward since these patients have begun to be examined in the lateral position by means of the $x$-rays. It can often be demonstrated very clearly that the cause of the abdominal distension is enormous dilatation of the bowel, and especially of the colon, with some kind of gas, and the name "aërocoly" has been applied to the condition. The gas is not air in the ordinary sense, for it consists largely of hydrogen and methane; these gases are produced as the result of putrefactive changes in proteid constituents within the bowel, and the relief afforded by the administration of a simple powder, containing some such ingredients as three grains of sodium bicarbonate and one of mercury and chalk, once, twice, or three times a day, would indicate that the correct treatment of the condition, in addition to the correction of any dietetic errors that may be obvious in the case, is by means of intestinal antiseptics of a mild kind, particularly small doses of mercury.

### A New Tropical Medicine Bulletin.

It is announced that the Managing Committee of the Sleeping Sickness Bureau has decided to publish, from the same offices as the *Sleeping Sickness Bureau Bulletin* is now published, a new Quarterly Bulletin dealing with the *Leishmania* group of diseases. Dr. C. M. Wenyon, Protozoologist to the London School of Tropical Medicine, will undertake the editorship of this new periodical. A list of references is now in preparation, and will constitute the first number. Those who wish to receive the new Bulletin should send their names to the Director, who will be glad to have copies of publications on this subject for the Bureau Library.

